# A Systematic Review of PTSD Prevalence and Trajectories in DSM-5 Defined Trauma Exposed Populations: Intentional and Non-Intentional Traumatic Events

**DOI:** 10.1371/journal.pone.0059236

**Published:** 2013-04-11

**Authors:** Patcho N. Santiago, Robert J. Ursano, Christine L. Gray, Robert S. Pynoos, David Spiegel, Roberto Lewis-Fernandez, Matthew J. Friedman, Carol S. Fullerton

**Affiliations:** 1 Uniformed Services University of the Health Sciences, Center for the Study of Traumatic Stress, Bethesda, Maryland, United States of America; 2 University of California Los Angeles, Department of Psychiatry and Biobehavioral Sciences, Los Angeles, California, United States of America; 3 Stanford University School of Medicine, Center on Stress and Health, Stanford, California, United States of America; 4 Columbia University, Department of Psychiatry, and the New York State Psychiatric Institute, New York, New York, United States of America; 5 Department of Veterans Affairs, National Center for Posttraumatic Stress Disorder, White River Junction, Vermont, and the Departments of Psychiatry and Pharmacology & Toxicology, Geisel School of Medicine at Dartmouth, Hanover, New Hampshire, United States of America; University of Pennsylvania, United States of America

## Abstract

**Objective:**

We conducted a systematic review of the literature to explore the longitudinal course of PTSD in DSM-5-defined trauma exposed populations to identify the course of illness and recovery for individuals and populations experiencing PTSD.

**Methods:**

We reviewed the published literature from January 1, 1998 to December 31, 2010 for longitudinal studies of directly exposed trauma populations in order to: (1) review rates of PTSD in the first year after a traumatic event; (2) examine potential types of proposed DSM-5 direct trauma exposure (intentional and non-intentional); and (3) identify the clinical course of PTSD (early onset, later onset, chronicity, remission, and resilience). Of the 2537 identified articles, 58 articles representing 35 unique subject populations met the proposed DSM-5 criteria for experiencing a traumatic event, and assessed PTSD at two or more time points within 12 months of the traumatic event.

**Results:**

The mean prevalence of PTSD across all studies decreases from 28.8% (range  = 3.1–87.5%) at 1 month to 17.0% (range  = 0.6–43.8%) at 12 months. However, when traumatic events are classified into intentional and non-intentional, the median prevalences trend down for the non-intentional trauma exposed populations, while the median prevalences in the intentional trauma category steadily increase from 11.8% to 23.3%. Across five studies with sufficient data, 37.1% of those exposed to intentional trauma develop PTSD. Among those with PTSD, about one third (34.8%) remit after 3 months. Nearly 40% of those with PTSD (39.1%) have a chronic course, and only a very small fraction (3.5%) of new PTSD cases appears after three months.

**Conclusions:**

Understanding the trajectories of PTSD over time, and how it may vary by type of traumatic event (intentional vs. non-intentional) will assist public health planning and treatment.

## Introduction

Longitudinal studies of responses to traumatic events document the course of illness and recovery in trauma-exposed populations confirming, as the Diagnostic and Statistical Manual (DSM) has written, that posttraumatic stress disorder has a variable course that can be acute or chronic, remitting after only three months, delayed after six months, or lasting for years. Other studies have longitudinally examined the effectiveness of treatment interventions [1], [2], which highlight the advantage of early intervention to shorten the time to remission of symptoms. Data from control groups in these intervention studies often also reveal the natural course of PTSD. Studies using DSM-IV criteria have followed subjects to examine the epidemiology of PTSD after disasters [3], [4], [5], [6], other traumatic events [7], [8], [9], and military deployment [10], [11], again finding substantial variability across different populations, traumatic events and community contexts. Knowing patterns of response after traumatic events can inform health system interventions after a disaster or traumatic event.

The proposed DSM-5 criteria highlight the importance of direct exposure as a specific category of traumatic experience and serve to narrow variation in the application of this criterion [12]. Experiences that meet the DSM-IV and proposed DSM-5 traumatic events criterion range from direct exposure, such as motor vehicle accidents, mud slides, and terrorist attack, to witnessing a traumatic event [13]. A number of studies have examined broad classifications of trauma exposures, including natural vs. human-made and intentional vs. non-intentional [14], [15], [16], [17]. The importance of differences between intentional and non-intentional traumatic events has been explored when examining treatment efficacy and attrition. Intentional traumas are those that involve the deliberate infliction of harm, and those exposed to intentional traumatic events had worse health outcomes than those who experienced harm that was inadvertent [18], [19], [20].

In order to better understand the course of PTSD during the first year after exposure, we conducted a systematic review of the empirical literature. We identified longitudinal studies reporting the prevalence of untreated PTSD in the same cohort or in a nationally representative sample, at two or more points in time within one year after direct exposure to a traumatic event that met the proposed DSM-5 criterion. We examined the longitudinal prevalence of PTSD in exposed populations, as well as the course of illness and recovery for individuals experiencing PTSD (early onset, later onset, chronicity, remission and resilience) in the first year after trauma exposure. This paper provides PTSD prevalence estimates, including the differences in prevalence between intentional and non-intentional traumas, which may inform our understanding of both prognosis and recovery, as well as have implications for public health treatment needs.

## Methods

### Search Criteria

We reviewed the published literature from January 1, 1998 to December 31, 2010 for longitudinal studies of populations directly exposed to traumatic events. We chose 1998 to begin our review in order to update the literature since the review by Breslau et al (1998). We used the DSM-5 criteria for direct exposure PTSD to define a traumatic event and included studies published in English that measured PTSD prevalence using validated measures at two or more time points within twelve months post-trauma. Because we sought to identify patterns in the natural course of responses and recovery, we excluded studies if the subjects received treatment or other interventions, unless data from a control group were available. In addition, studies were excluded if they failed to meet the one-month duration criterion, if they measured only PTSD symptoms and not disorder, if the population of interest was children or adolescents under 18 years old, or if the study identified pregnancy or childbirth as a traumatic event (unless it was specified as a complicated or extraordinary event such as miscarriage). Finally, studies were excluded if the PTSD prevalence was not reported directly or was reported in a way that could not be calculated.

Our search strategy for this review involved three stages. First, we used PubMed and PILOTS databases to find abstracts using keyword combinations that included PTSD and each of the following: ‘longitudinal’, ‘acute’ and ‘chronic’, and key authors known to have conducted extensive research on the course of PTSD. Second, the citations were cross-referenced to eliminate duplicates prior to reviewing abstracts. Third, 2537 unique abstracts were reviewed. Those that explicitly stated inclusion criteria or provided information suggesting that the article may meet inclusion criteria were marked for further review.

### Analysis

In some studies, the desired information (prevalence of PTSD in the same cohort or in a representative sample at two or more time points) was not directly presented in the article. When possible, that information was calculated using other data presented in the article. In some cases, approximations were used to estimate time. For example, if a range of 4–8 months was provided for a time point, the midpoint (6 months) was used as the time point for the purpose of examining the course of illness for PTSD. Different articles with the same subject populations were combined as single studies. We grouped the data for each study into categories of 1, 3, 6, and 12 months post-trauma to allow comparison across studies and over time. Because the data are not symmetrically distributed, medians better represent the average values and were calculated at each of these time points.

All of the studies met the proposed DSM-5 criterion A. That is, the subject experienced “…one of the following event(s): death or threatened death, actual or threatened serious injury, or actual or threatened sexual violence...” [12]. To better understand the relationship between the nature of the traumatic event and the course of PTSD, we classified the traumatic events into either intentional (e.g., assault, war) or non-intentional (e.g., earthquake, motor vehicle accident). This classification yielded 14 intentional and 21 non-intentional traumatic event studies.

Of the 2537 identified articles, 58 articles representing 35 unique subject populations met criteria of being assessed for PTSD at two or more time points within 12 months of a traumatic event and met the proposed DSM-5 criteria for experiencing a direct traumatic event. Those 35 populations were analyzed for this review (Table S1). The trauma exposures include: motor vehicle/plane crash (N = 8 studies), assault (N = 4), terrorism (N = 7), war as a combatant or civilian (N = 3), natural disaster (N = 4), severe injury warranting a hospital visit (N = 7), and serious, life-threatening medical condition (N = 2). For our analysis of trajectories, we identified studies that included a report of PTSD assessed in individual subjects in at least two time points within a year. This made possible identifying the course of PTSD in individual subjects. Among our examined studies, five of the 14 intentional trauma studies included sufficient information to examine the PTSD trajectory of individual subjects, allowing for calculation of the trajectories of PTSD within individuals. These studies reported PTSD at two different time points, where Time 1 was 1 to 1.5 months (4–6 weeks) post-trauma, and Time 2 was 3 to 12 months post trauma. Data was not sufficient to perform parallel analyses for non-intentional trauma. We calculated the percent of individuals who were never diagnosed with PTSD (were resilient), achieved remission during the first year, had a late onset of PTSD, and those who experienced chronic symptoms of PTSD.

## Results

### Longitudinal Prevalence of PTSD by Trauma Type

We examined medians of the PTSD prevalence at each time point (Table 1). In general, the trend of the means and medians are similar. The median prevalence of PTSD across all studies decreases from 28.8% (range  = 3.1–87.5%) at 1 month to 17.0% (range  = 0.6–43.8%) at 12 months (see Table 1). There is a drop in PTSD median prevalence between month 1 (28.8%) and month 3 (17.8%), after which the median prevalence appears to stabilize. These prevalences are similar to previously published rates across different types of traumatic events [14], [15], [21], [22].

**Table 1:** Mean and median prevalence of PTSD in exposed populations meeting DSM-5 Direct Experiencing criteria (N = 35 studies). 1

	DSM-5-Experiencing (N = 35 studies)		Intentional Injury or Trauma (N = 14 Studies)		Non-Intentional Injury or Trauma (N = 21 Studies)	
Months post-trauma	Median Prevalence	Mean Prevalence	Median Prevalence	Mean Prevalence	Median Prevalence	Mean Prevalence
	% (range)	% (sd)	% (range)	% (sd)	% (range)	% (sd)
1	28.8 (3.1–87.5)	25.4 (20.2)	11.8 (3.1–87.5)	23.6 (26.2)	30.1 (16.7–35.1)	28.0 (7.0)
3	17.8 (1.6–44.8)	18.8 (11.1)	17.1 (1.7–44.8)	18.9 (14.9)	17.8 (8.0–39.2)	18.8 (8.8)
6	14.9 (0.6–40.3)	16.1 (11.4)	19.0 (0.6–40.3)	18.3 (13.6)	12.9 (3.1–33.3)	14.4 (9.8)
12	17.0 (0.6–43.8)	17.7 (10.8)	23.3 (2.6–43.8)	23.1 (13.6)	14.0 (2.2–28.6)	14.8 (8.2)

1 The DSM-5-Experiencing category was based on meeting proposed DSM-5 criteria for direct experience of a traumatic event. Assessment points in studies were grouped into categories of 1, 3, 6, or 12 months post-trauma based on closest match to the actual assessment time point.

**Figure 1:**
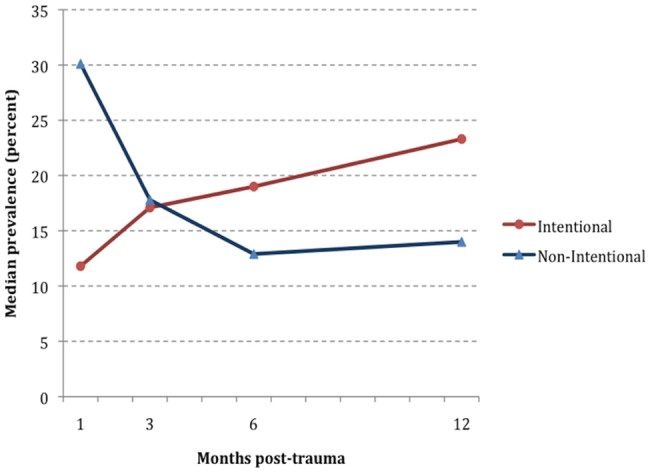
Median prevalence of PTSD in DSM-5-Experiencing categories of intentional and non-intentional trauma (N = 14 and 21 studies, respectively).

Examination of PTSD prevalence across time (1, 3, 6 and 12 months) in the different traumatic event categories shows some differences by category (see Table 1). The trend in PTSD prevalence among those exposed to a non-intentional trauma is decreasing over time (30.1% at month 1 and 14.0% at month 12). The intentional trauma group shows a different course with the median prevalences increasing from 11.8% to 23.3%. This is particularly visible in the graphs of the median prevalence over time (Figure 1).

### Trajectory of PTSD

Of individuals exposed to intentional traumatic events, a median of 37.1% (range 6.5–87.5%) developed PTSD in the first year after exposure (Table 2). Therefore, 62.9% (range 12.5–93.5%) never developed PTSD. Among the exposed, a median of 12.9% (range 1.7–43.8%) had PTSD only at Time 1. This represents 34.8% of those ever diagnosed. Similarly, a median of 14.5% had PTSD at both Time 1 and Time 2 (39.0% of those diagnosed with PTSD) and 1.3% had PTSD onset after Time 1 (3.5% of those diagnosed with PTSD).

**Table 2:** Individual trajectories of PTSD prevalence in DSM-5-Direct Experiencing category with intentional trauma exposure (N = 5 studies).^2^

		Trajectory			
		Never diagnosed	Remission	Chronicity	Later onset
Study	PTSD Ever	PTSD Never	T1 only PTSD	PTSD All Times	PTSD onset after T1
	N (%)	N (%)	N (%)	N (%)	N (%)
Vojvoda, et al (2008) [30]	14 (87.5%)	2 (12.5%)	7 (43.8%)	7 (43.8%)	0 (0.0%)
Roy-Byrne, et al (2004) [31]	14 (60.9%)	9 (39.1%)	7 (30.4%)	7 (30.4%)	0 (0.0%)
Johnson, et al (2002) [32]	5 (6.5%)	72 (93.5%)	3 (3.9%)	1 (1.3%)	1 (1.3%)
North, et al (2001, 1997) [33], [34]	46 (37.1%)	78 (62.9%)	16 (12.9%)	18 (14.5%)	12 (9.7%)
Grieger, et al (2006) [10]	34 (14.0%)	209 (86.0%)	4 (1.7%)	4 (1.7%)	26 (10.7%)
Median Prevalence	37.1%	62.9%	12.9%	14.5%	1.3%
Mean Prevalence	41.1%	58.9%	18.5%	18.3%	4.3%

2 Studies are those that reported prevalences for each possible trajectory of PTSD diagnosis at two or more time points within 12 months post-trauma. T1 indicates a time period 1 to 1.5 months post-trauma and T2 indicates a time period 3 to 12 months post trauma.

## Discussion

Overall, we found that when we separated intentional and non-intentional trauma, two population courses were suggested for the prevalence of PTSD across time. The prevalence of PTSD increased over time after intentional traumatic events and decreased after non-intentional traumatic events, indicating the overall public health burden of PTSD was greater in those exposed to intentional traumatic events. Of note, at one month, non-intentional traumatic events had a higher median prevalence of PTSD than intentional trauma. For public health planning, recognizing that the type of the traumatic event may mean a different natural course of the disorder can affect resource planning and treatment.

In order to examine the trajectory of PTSD, we examined studies of populations that had directly experienced traumatic events as listed in DSM-5, which are the most studied in the existing literature. Other proposed A criteria (i.e., witnessing an event, learning of an event, or experiencing repeated indirect exposures) may yield other courses and trajectories. There were only a small number of studies that met our criteria for addressing individual trajectories of PTSD over time, and all of these were for intentional traumatic events. The trajectories of PTSD after intentional traumatic events show wide variability, but on average, approximately one-third of those exposed developed PTSD in the first year. Importantly, nearly two-thirds did not. Among those with PTSD, about one third remitted within 3 months, while 39% of those with PTSD had a chronic course, supporting the notion that PTSD can spontaneously resolve or continue as a persistent disorder. Onset of PTSD after 3 months represented a small fraction (3.5%) of the total PTSD cases. Nevertheless, a “delayed expression” specifier to the diagnosis is supported by the data.

Our study did not examine predictors or risk factors that may modulate the different courses of PTSD in populations related to intentional and non-intentional exposures. These include genetic, dispositional, and recovery environment factors. Specifically, the types of populations exposed to intentional and non-intentional traumas can differ substantially in characteristics and contextual issues, due to socioeconomic factors, employment, cultural differences, and available resources. These factors can substantially contribute to the different courses of PTSD. A recent study of PTSD that examined multiple studies across different disaster types similarly found differences in rates of PTSD between intentional and non-intentional disasters (26% in “intentionally caused” disasters compared to 10% and 16% in “technological” and “natural” disasters, respectively). However, the differences were not present after controlling for pre-disaster conditions and sample characteristics [22]. This is consistent with our findings and suggests that the difference in outcomes between intentional and non-intentional traumatic events is mediated by the severity of exposure, the characteristics of the populations exposed, and the recovery environment.

Few studies have followed participants for more than a year and with more than two assessments. This is unfortunate since it limits what one can investigate. For example, in the 20-year longitudinal study of Israeli veterans of the Lebanon War, which delineates the diagnostic patterns of 214 veterans at 1, 2, 3, and 20 years [23], a fluctuating course of PTSD (e.g., a variable pattern of remissions and relapses) was detected along with the remitting, persistent and delayed courses observed in the present study. In addition, studies of different disaster types and across cultures may yield different PTSD trajectories [24], [25], [26].

This study is limited by the relatively few studies available with longitudinal data. Our study is also focused only on directly experienced traumatic events. The broad set of categories originally delineated by the 1996 Detroit-area survey studying trauma and PTSD in the community [14] grouped events as “assault,” “other injury or shocking experience,” “learning about trauma to others,” or “sudden unexpected death of a close friend or relative.” The latter two categories exemplify indirect traumatic exposure and, therefore, were not included in this review. In contrast, the two former categories map onto our broader terms of intentional and non-intentional traumatic events so they were included. This study also examined the literature from a specific 13-year period when the DSM-IV definition of PTSD was in use. We considered possible bias in the data in the studies we examined. Psychiatric epidemiology studies consistently report a lifetime prevalence of PTSD of approximately 8% [15], [16], [17], however, post-disaster rates of PTSD vary widely [27], [28], [29], and are similar to those found here. One could expect measurement bias in our study because of the different instruments used to obtain data in the different studies. However, this would not substantially affect the overall patterns found in this study, as the same instruments were used across time in individual studies. To further explore our finding of an increasing rate of PTSD in intentional traumas with an overall decreasing rate in non-intentional traumas, more detail on traumatic event characteristics, the degree of exposure and the context would be helpful. Systematically including this information in future studies will be required to address these issues.

Our results indicate that the type of events, whether being intentional or non-intentional, appear to affect both the prevalence of PTSD and its trajectories over time. Our findings reinforce the importance of longitudinal research in understanding the course, prognosis, and severity of PTSD. Such information is valuable for planning and implementing appropriate individual and population level interventions.

## Supporting Information

Table S1 Appendix: Summary of studies measuring and reporting PTSD prevalence at two or more time points within 12 months post-trauma.
(DOC)
