# Supplementary material for: A Systematic Review of PTSD Prevalence and Trajectories in DSM-5 Defined Trauma Exposed Populations: Intentional and Non-Intentional Traumatic Events
Source: PLoS One. 2013 Apr 11;8(4):e59236. doi: 10.1371/journal.pone.0059236 (PMC3623968; doi:10.1371/journal.pone.0059236)
Supplement: Table S1 — Appendix: Summary of studies measuring and reporting PTSD prevalence at two or more time points within 12 months post-trauma. (DOC) [file pone.0059236.s001.doc]

**Table S1. Appendix: Summary of studies measuring and reporting PTSD prevalence at two or more time points within 12 months post-trauma.**

|  |  |  |  |  | **Prevalence** | | | | | | | | | |
| --- | --- | --- | --- | --- | --- | --- | --- | --- | --- | --- | --- | --- | --- | --- |
| **Study** | **Trauma** | **Intentional Trauma^1^** | **N** | **Measure** | **T1 (time in months)** | **%** | **T2** | **%** | **T3** | **%** | **T4** | **%** | **T5** | **%** |
| Conejo-Galindo et al., 2007 [1] | Madrid terror attack | I | 56 | MINI | 1 | 35.7 | 6 | 34.1 | 12 | 28.6 |  |  |  |  |
| Essizoglu et al., 2009 [2] | Turkey Terror Attack | I | 216 | Basoglu Scale | 1 | 12.5 | 3 | 9.6 |  |  |  |  |  |  |
| Galea et al., 2003 [3] | World Trade Center Attack | I | 1008 | National Women’s Study Questions | 1 | 7.5 | 4 | 1.7 | 6 | 0.6 |  |  |  |  |
| Grieger et al., 2006 [4] | Combat | I | 613 | PTSD Checklist | 1 | 4.2 | 4 | 12.2 | 7 | 12.0 |  |  |  |  |
| Johansen et al., 2007 [5] | Non-domestic Assault | I | 143 | PTSS-10 | 2 | 28.6 | 5 | 24.3 | 14 | 27.1 |  |  |  |  |
| Johnson et al., 2002 [6] | Mass Shooting | I | 80 | DIS | 1 | 5.0 | 6 | 3.8 | 12 | 2.6 | 24 | 7.8 | 36 | 2.6 |
| Marchand et al., 2006 [7] | Armed Robbery | I | 42 | SCID | 1 | 3.1 | 3 | 3.1 |  |  |  |  |  |  |
| North et al., 2001 [8] | Mass Shooting | I | 136 | DIS | 3.5 | 29.0 | 12 | 17.0 | 36 | 19.0 |  |  |  |  |
| Panos et al., 2004 [9] | Hostage Situation | I | 172 | IES | 6 | 23.3 | 12 | 21.3 | 24 | 18.1 | 60 | 13.2 | 84 | 4.6 |
| Roth et al., 2006 [10] | War, civilian non-combat | I | 218 | HTQ | 3 | 44.8 | 6 | 40.3 | 18 | 80.0 |  |  |  |  |
| Roy-Byrne et al., 2004 [11] | Assault | I | 56 | CAPS | 1 | 36.8 | 3 | 21.9 |  |  |  |  |  |  |
| Sungur et al., 2001 [12] | Sivas Terror Attack | I | 79 | IES | 1.5 | 32.6 | 6 | 19.0 | 12 | 25.3 | 18 | 30.4 |  |  |
| Vojvoda et al., 2008 [13] | War, civilian non-combat | I | 28 | PSS | 1 | 87.5 | 12 | 43.8 | 42 | 25.0 |  |  |  |  |
| Walters et al., 2007 [14] | Assault | I | 562 | DTS | 1 | 11.0 | 6 | 7.7 |  |  |  |  |  |  |
| Bacher et al., 2005 [15] | MVA | NI | 144 | CAPS | 1 | 20.0 | 4 | 18.4 |  |  |  |  |  |  |
| Chung et al., 2009 [16] | Serious Injury (Hospital visit) | NI | 196 | PDS | 3 | 10.2 | 6 | 4.1 |  |  |  |  |  |  |
| Epstein et al., 1998 [17] | Plane Crash | NI | 355 | SCID | 6 | 7.5 | 12 | 12.1 | 18 | 7.3 |  |  |  |  |
| Hepp et al., 2008[18] | Severe Injury (Hospital visit) | NI | 121 | CAPS | 6 | 3.3 | 12 | 2.2 | 36 | 4.4 |  |  |  |  |
| Jeavons et al., 2000 [19] | MVA | NI | 72 | PTSD-Interview | 3 | 8.3 | 6 | 8.0 | 12 | 8.6 |  |  |  |  |
| Jenewin et al., 2009 [20] | Serious Injury (Hospital visit) | NI | 323 | CAPS | 6 | 3.1 | 12 | 5.6 |  |  |  |  |  |  |
| Jones et al., 2007 [21] | MVA | NI | 131 | PSS | 1.5 | 22.8 | 3 | 17.6 |  |  |  |  |  |  |
| Karamustafalioglu et al, 2006 [22] | Earthquake | NI | 464 | PTSD Self-test | 2 | 30.2 | 8 | 26.9 | 19 | 10.6 |  |  |  |  |
| Kessler et al., 2008 [23] | Hurricane | NI | 815 | TSQ | 6.5 | 14.9 | 12 | 20.9 |  |  |  |  |  |  |
| Kuhn et al., 2006 [24] | MVA | NI | 42 | CAPS | 1 | 16.7 | 3 | 10.8 | 6 | 10.8 |  |  |  |  |
| Malta et al., 2002 [25] | MVA | NI | 158 | CAPS | 2.5 | 39.2 | 8.5 | 18.6 | 14.5 | 14.4 |  |  |  |  |
| Mayou et al., 2002 [26] | MVA | NI | 1441 | PSS | 3 | 23.1 | 12 | 16.5 | 36 | 11.0 |  |  |  |  |
| McKibben et al., 2008 [27] | Burn injury | NI | 178 | DTS | 1 | 35.1 | 6 | 33.3 | 12 | 28.6 | 24 | 25 | 4 |  |
| Nielson et al., 2003 [28] | Spinal Cord Lesion | NI | 85 | HTQ | 2.5 | 23.2 | 5 | 22.5 | 9 | 23.7 |  |  |  |  |
| Norris et al. 2004 [29] | Flood | NI | 561 | CIDI | 6 | 23.9 | 12 | 18.0 | 18 | 10.7 | 24 | 11.1 |  |  |
| O’Donnell et al., 2005 [30] | Serious injury (hospital visit) | NI | 363 | CAPS | 3 | 8.6 | 12 | 10.4 |  |  |  |  |  |  |
| Phelps et al., 2008 [31] | Serious Injury (Amputation) | NI | 130 | PCL | 6 | 22.9 | 12 | 26.0 |  |  |  |  |  |  |
| Shalev et al., 1998 [32] | Serious Injury (hospital visit) | NI | 211 | CAPS | 1 | 30.1 | 4 | 17.9 |  |  |  |  |  |  |
| Sheldrick et al., 2006 [33] | MI (hospital visit) | NI | 68 | DTS | 1.5 | 31.0 | 3 | 16.0 |  |  |  |  |  |  |
| Ursano et al., 1999 [34] | MVA | NI | 122 | SCID | 1 | 34.4 | 3 | 25.3 | 6 | 18.2 | 9 | 17.4 | 12 | 14.0 |
| Wang et al., 2000 [35] | Earthquake | NI | 181 | CIDI | 3 | 14.4 | 9 | 17.8 |  |  |  |  |  |  |

I = Intentional Trauma; NI = Non-Intentional Trauma

References

1. Conejo-Galindo J, Medina Ó, Fraguas D, Terán S, Sainz Cortón E, et al. (2007) Psychopathological sequelae of the 11 March terrorist attacks in Madrid. European Archives of Psychiatry and Clinical Neuroscience 258: 28-34.
2. Essizoglu A, Yasan A, Bulbul I, Onal S, Yildirim EA, et al. (2009) [Factors affecting the diagnosis of post-traumatic stress disorder after a terrorist attack]. Turk Psikiyatri Derg 20: 118-126.
3. Galea S, Vlahov D, Resnick H, Ahern J, Susser E, et al. (2003) Trends of probable post-traumatic stress disorder in New York City after the September 11 terrorist attacks. Am J Epidemiol 158: 514-524.
4. Grieger TA, Cozza SJ, Ursano RJ, Hoge CW, Martinez PE, et al. (2006) Posttraumatic stress disorder and depression in battle-injured soldiers. American Journal of Psychiatry 163: 1777-1783.
5. Johansen VA, Wahl AK, Eilertsen DE, Weisæth L, Hanestad BR (2007) The predictive value of post-traumatic stress disorder symptoms for quality of life: a longitudinal study of physically injured victims of non-domestic violence. Health and Quality of Life Outcomes 5: 26.
6. Johnson SD, North CS, Smith EM (2002) Psychiatric disorders among victims of a courthouse shooting spree: a three-year follow-up study. Community Mental Health Journal 38: 181-194.
7. Marchand A, Guay Sp, Boyer R, Iucci S, Martin A, et al. (2006) A randomized controlled trial of an adapted form of individual critical incident stress debriefing for victims of an armed robbery. Brief Treatment and Crisis Intervention 6: 122-129.
8. North CS (2001) The course of post-traumatic stress disorder after the Oklahoma City bombing. Military Medicine 166: 51-52.
9. Panos A, Panos PT, Dulle P (2004) A 10-year clinical case study to an incident of workplace violence. Journal of Emotional Abuse 4: 23-47.
10. Roth Gr, Ekblad S, Ågren H (2006) A longitudinal study of PTSD in a sample of adult mass-evacuated Kosovars, some of whom returned to their home country. European Psychiatry 21: 152-159.
11. Roy-Byrne PP, Russo JE, Michelson E, Zatzick DF, Pitman RK, et al. (2004) Risk factors and outcome in ambulatory assault victims presenting to the acute emergency department setting: implications for secondary prevention studies in PTSD. Depression and Anxiety 19: 77-84.
12. Sungur M, Kaya B (2001) The onset and longitudinal course of a man-made post-traumatic morbidity: survivors of the Sivas disaster. International Journal of Psychiatry in Clinical Practice 5: 195-202.
13. Vojvoda D, Weine SM, McGlashan TH, Becker DF, Southwick SM (2008) Posttraumatic stress disorder symptoms in Bosnian refugees 3 1/2 years after resettlement. Journal of Rehabilitation Research and Development 45: 421-426.
14. Walters JTR, Bisson JI, Shepherd JP (2007) Predicting post-traumatic stress disorder: validation of the Trauma Screening Questionnaire in victims of assault. Psychological Medicine 37: 143-150.
15. Bachar E, Hadar H, Shalev AY (2005) Narcissistic vulnerability and the development of PTSD: a prospective study. Journal of Nervous and Mental Disease 193: 762-765.
16. Chung MC, McKee KJ, Austin C, Barkby H, Brown H, et al. (2009) Posttraumatic stress disorder in older people after a fall. Int J Geriatr Psychiatry 24: 955-964.
17. Epstein RS, Fullerton CS, Ursano RJ (1998) Posttraumatic stress disorder following an air disaster: a prospective study. American Journal of Psychiatry 155: 934-938.
18. Hepp U, Moergeli H, Büchi S, Bruchhaus-Steinert H, Kraemer B, et al. (2008) Post-traumatic stress disorder in serious accidental injury: 3-year follow-up study. British Journal of Psychiatry 192: 376-383.
19. Jeavons S (2000) Predicting who suffers psychological trauma in the first year after a road accident. Behaviour Research and Therapy 38: 499-508.
20. Jenewein J, Wittmann L, Moergeli H, Creutzig J, Schnyder U (2009) Mutual influence of posttraumatic stress disorder symptoms and chronic pain among injured accident survivors: a longitudinal study. J Trauma Stress 22: 540-548.
21. Jones C, Harvey AG, Brewin CR (2007) The organisation and content of trauma memories in survivors of road traffic accidents. Behaviour Research and Therapy 45: 151-162.
22. Karamustafalioglu OK, Zohar J, GüveliM, Gal G, Bakim B, et al. (2006) Natural course of posttraumatic stress disorder: a 20-month prospective study of Turkish earthquake survivors. Journal of Clinical Psychiatry 67: 882-889.
23. Kessler RC, Galea S, Gruber MJ, Sampson NA, Ursano RJ, et al. (2008) Trends in mental illness and suicidality after Hurricane Katrina. Mol Psychiatry 13: 374-384.
24. Kuhn E, Blanchard EB, Fuse T, Hickling EJ, Broderick J (2006) Heart rate of motor vehicle accident survivors in the emergency department, peritraumatic psychological reactions, ASD, and PTSD severity: a 6-month prospective study. Journal of Traumatic Stress 19: 735-740.
25. Malta LS, Blanchard EB, Taylor AE, Hickling EJ, Freidenberg BM (2002) Personality disorders and posttraumatic stress disorder in motor vehicle accident survivors. Journal of Nervous and Mental Disease 190: 767-774.
26. Mayou RA, Bryant B (2002) Outcome 3 years after a road traffic accident. Psychological Medicine 32: 671-675.
27. McKibben JBA, Bresnick MG, Askay SAW, Fauerbach JA (2008) Acute stress disorder and posttraumatic stress disorder: a prospective study of prevalence, course, and predictors in a sample with major burn injuries. Journal of Burn Care and Research 29: 22-35.
28. Nielsen MS (2003) Crisis support and coping as mediators of well-being in persons with spinal cord lesion. Journal of Clinical Psychology in Medical Settings 10: 91-99.
29. Norris FH, Murphy AD, Baker CK, Perilla JL (2004) Postdisaster PTSD over four waves of a panel study of Mexico's 1999 flood. Journal of Traumatic Stress 17: 283-292.
30. O'Donnell ML, Creamer MC, Elliott P, Atkin C (2005) Health costs following motor vehicle accidents: the role of posttraumatic stress disorder. Journal of Traumatic Stress 18: 557-561.
31. Phelps LF, Williams RM, Raichle KA, Turner AP, Ehde DM (2008) The importance of cognitive processing to adjustment in the 1st year following amputation. Rehabilitation Psychology 53: 28-38.
32. Shalev AY, Freedman SA, Peri T, Brandes D, Sahar T, et al. (1998) Prospective study of posttraumatic stress disorder and depression following trauma. American Journal of Psychiatry 155: 630-637.
33. Sheldrick R, Tarrier N, Berry E, Kincey J (2006) Post-traumatic stress disorder and illness perceptions over time following myocardial infarction and subarachnoid haemorrhage. Br J Health Psychol 11: 387-400.
34. Ursano RJ, Fullerton CS, Epstein RS, Crowley B, Kao T-C, et al. (1999) Acute and chronic posttraumatic stress disorder in motor vehicle accident victims. American Journal of Psychiatry 156: 589-595.
35. Wang X, Gao L, Shinfuku N, Zhang H, Zhao C, et al. (2000) Longitudinal study of earthquake-related PTSD in a randomly selected community sample in North China. American Journal of Psychiatry 157: 1260-1266.
